# The Usefulness of the Navigation System to Reconstruct Orbital Wall Fractures Involving Inferomedial Orbital Strut

**DOI:** 10.3390/jcm12154968

**Published:** 2023-07-28

**Authors:** Tae Hwan Park

**Affiliations:** Department of Plastic and Reconstructive Surgery, Dongtan Sacred Heart Hospital, Hallym University College of Medicine, Hwaseong 18450, Republic of Korea; plasticpth@gmail.com; Tel.: +82-031-8086-2640; Fax: +82-031-8086-2641

**Keywords:** facial bone, trauma, orbital fracture, orbital floor, medial wall, navigation, enophthalmos

## Abstract

Background: Little attention has been paid to combined orbital floor and medial wall fractures with the involvement of the inferomedial orbital strut. Managing this particular fracture can prove challenging. However, various innovative techniques have been introduced to assist with the process. Our study focuses on sharing our approach to orbital wall reconstruction using navigation guidance and titanium-reinforced porous polyethylene plates, specifically cases involving the inferomedial orbital strut. We believe that implementing a navigation system can effectively lead surgeons to the fracture site with utmost safety. Also, we hypothesized that this navigation system is beneficial to use singe fan titanium-reinforced porous polyethylene plates with orbital wall fractures involving IOS while minimizing possible complications. Methods: We retrospectively reviewed 131 patients with medial orbital wall and orbital floor fractures with or without combined other facial bone fractures who underwent orbital wall reconstruction by a single surgeon from May 2021 to May 2023. Amongst, we identified fourteen orbital wall fractures involving the inferomedial orbital strut. We used a subciliary incision as the only approach method for performing titanium-reinforced porous polyethylene plates for navigation-guided orbital wall reconstruction. Patients were followed up for at least three months. Results: All cases were effectively resolved using titanium-reinforced porous polyethylene plates. There were no complications during the patient’s complete recovery, confirmed clinically and radiologically. Based on the serial CT results, it was discovered that implanted titanium-reinforced porous polyethylene plates successfully covered the defect. Conclusion: Based on our retrospective analysis, it has been determined that among the 131 recorded cases of orbital fractures, 14 of them (or 10.7%) involved the inferomedial orbital strut. Navigation-guided reduction using titanium-reinforced porous polyethylene (TR-PPE) plates can lead to predictable, reliable, and excellent outcomes for treating orbital fractures involving the inferomedial orbital strut without complications.

## 1. Introduction

Facial fractures are frequently encountered in clinical practice, with orbital blow-out fractures being a common occurrence [1]. These fractures typically involve injury to the orbital floor or medial wall [2]. However, in certain instances, more extensive damage can occur in the inferomedial region, resulting in what is referred to as an inferomedial blow-out fracture [3]. This particular fracture type encompasses the orbital floor, medial wall, and intervening inferomedial orbital strut (IOS) [4]. The consequences of such a fracture can be significant, as it often leads to substantial herniation of orbital fat, causing expansion of the orbit and displacement of the globe [5]. Restoring this fracture anatomically can be difficult due to limited structural support [6]. Nevertheless, it is imperative to perform anatomical reconstruction of the orbit to mitigate the risk of serious complications, including diplopia, visual impairment, and enophthalmos [7].

Additionally, there are wide available materials to reconstruct the orbital wall. Titanium-reinforced porous polyethylene plates [8] are preferred over other materials for reconstructing the extensive orbital wall fractures due to their ability to be securely fixed with screws and accurately evaluated in the postoperative result with a titanium silhouette on the CT scan. Due to its unique properties, titanium-reinforced porous polyethylene (TPE) has become a popular choice for orbital reconstruction procedures. There are some pros and cons associated with its use [9]: Titanium-reinforced porous polyethylene is biocompatible when implanted, with a low risk of adverse reactions or tissue rejection. TPE’s porous structure enables tissue growth, increasing vascularization and integration with nearby bones and soft tissues. This can enhance the stability and long-term success of the implant. TPE is highly adaptable and can be customized to suit the individual anatomical needs of the patient. Surgeons can sculpt the implant during the procedure to achieve optimal results. TPE does not affect imaging techniques like X-rays or CT scans, as it is radiolucent. This allows for postoperative monitoring and assessment of the reconstructed area without the need for implant removal. Titanium-reinforced porous polyethylene is relatively lightweight compared to other materials used in orbital reconstruction, such as titanium or autogenous bone grafts. This can reduce the strain on surrounding tissues and improve patient comfort.

While TPE has good initial stability, long-term outcomes are still being evaluated. Over time, the porous structure may degrade or lose its mechanical integrity, potentially leading to implant failure or complications. TPE is less robust than materials like titanium. Therefore, it may not be suitable for cases requiring significant load-bearing support, such as in extensive orbital fractures or defects. The porous nature of TPE can make revision surgeries more challenging. Removing or modifying the implant may require additional surgical techniques or tools to address tissue ingrowth, which can increase surgical complexity. Titanium-reinforced porous polyethylene implants can be more expensive than other materials. The additional cost may limit its accessibility in certain healthcare settings or for patients with financial constraints.

We hypothesized that utilizing titanium-reinforced porous polyethylene plates through a standard subciliary incision alone could lead to successful anatomical reconstruction for extensive orbital wall fractures involving IOS, while providing an aesthetically pleasing appearance.

## 2. Patients and Methods

This study was approved by the Institutional Review Board of our institution and conducted in accordance with the Declaration of Helsinki. Treatment consent was obtained from all patients. 

In this retrospective study, we evaluated the medical records, and 3D facial Computed Tomography (CT) scans of patients with pure orbital wall fractures involving IOS between May 2021 and May 2023. Facial CT scans confirmed the clinical diagnosis of pure medial orbital wall fractures or pure inferomedial orbital wall fractures involving IOS. We assessed the range of motion of eyeball movement, and the degree of enophthalmos was measured using the Keeler exophthalmometer. Recently, the iPlan CMF software (version 3.0) has been used as described [10]. Before measurement, CT data were used to construct a 3D coordinate system based on the exact craniofacial midsagittal plane, and the Frankfort horizontal plane was adjusted to be parallel to the horizontal plane. On the axial slice with the largest diameter of the eye globe, a baseline was drawn from the anterior point of the lateral orbital rim of the uninjured side, perpendicular to the midsagittal line. The globe projection was considered as the distance from the most prominent point of the cornea to the baseline. Furthermore, the difference in globe projection between the injured side and the uninjured side orbital was defined as the degree of enophthalmos. We gathered data for preoperative assessment, postoperative assessment, and final follow-up. Orbital volume was also evaluated with iPlan CMF. The software settings allowed for the orbital cavity of the damaged and contralateral orbits to be recorded under the soft tissue window. The range of the orbital cavity could be distinguished with the “auto segmentation” function of the software and displayed in 3D format. The orbital outline could be manually adjusted if required before the software automatically calculated the volume.

### 2.1. The Inclusion Criteria

1. Diagnosis of pure medial orbital wall fracture involving IOS confirmed by preoperative facial CT scans or diagnosis of inferomedial orbital wall fracture involving IOS confirmed by preoperative facial CT scans.
2. Postoperative evaluation, including clinical outcomes and radiological examination, including facial CT scans within three months postoperatively.

### 2.2. The Exclusion Criteria

1. Adjacent combined fractures requiring surgical reduction such as fracture of the zygomaticomaxillary complex and nasal bone fracture.
2. No available preoperative or postoperative 3D CT scans
3. No postoperative evaluation within three months postoperatively

### 2.3. Surgical Procedure

A single surgeon (THP) performed all surgical operations. Before the surgery, based on CT scan imaging, we measured the defect area and predicted the implant size we should use. Then, we approached the medial orbital wall or inferomedial wall fractures involving IOS with a subciliary incision alone. After fully exposing the lesion after checking the extent of the fracture with a navigation system (Supplementary Figures S1 and S2), the fracture depth and approximate width and length of the bony defect were measured with a paper ruler. The navigation was set by a simulation with an error rate of less than 1 (Fiagon Navigation System, Fiagon GmbH, Neuendorfstraße 23b D-16761 Hennigsdorf Germany).

Then, a trimmed single titanium-reinforced porous polyethylene (TR-PPE) plate was prepared and reshaped to correspond to the measured width and length. The implant was attached to the defect without injuring the inferior oblique muscle. After confirming the correct placement of the implant, it was fixed to the inferior orbital rim with a single absorbable (SCR-1220 1.5 × 4 mm Screw Ring, Inion, Finland) or a metal screw (Ø 1.3 mm Micro screws (non-locking), Jeil Medical, Seoul, Republic of Korea) (Supplementary Figure S3).

Depending on the depth of the medial orbital wall fracture, we additionally used folded ADM or trimmed absorbable mesh plate to cover the uppermost part of the medial wall fracture to prevent postoperative enophthalmos. Finally, a forced duction test was performed. The closure was performed meticulously, layer by layer.

## 3. Results

From May 2021 to March 2023, we treated eight cases of medial orbital wall involving IOS and six cases of inferomedial wall fracture involving IOS. Among 14 patients in total, nine patients were successfully reconstructed with a trimmed single titanium-reinforced porous polyethylene (TR-PPE) plate alone, while the remaining five patients recieved reconstruction with additional use of cross-linked ADM or absorbable mesh with significant improvement clinically and radiologically. Out of the 14 patients, three experienced a positive oculocardiac reflex. Two cases out of six inferomedial orbital walls involving IOS (33.3%) had oculocardiac reflex.

Baseline patient characteristics are presented in Table 1.

Representative cases are shown in Cases 1 through 4.

### 3.1. Case 1

**Figure 1:**
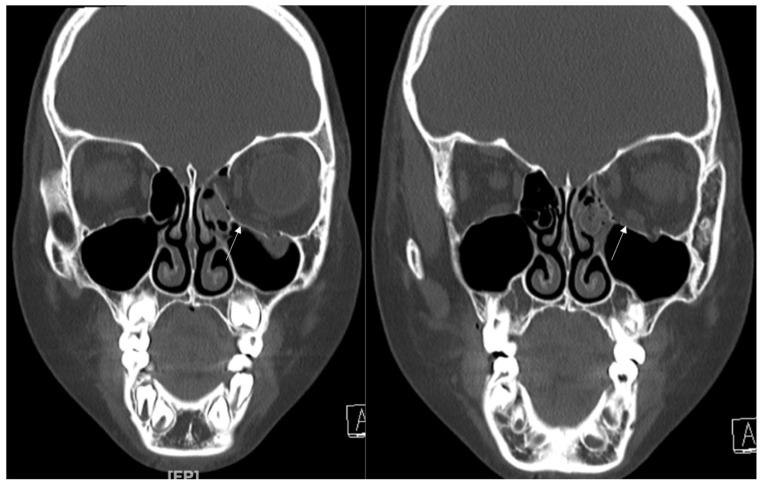
Case 1. Preoperative facial CT scan (coronal view) of the 8-year-old male patient with left inferomedial orbital wall fracture. The fracture sites were designated with white arrows.

**Figure 2:**
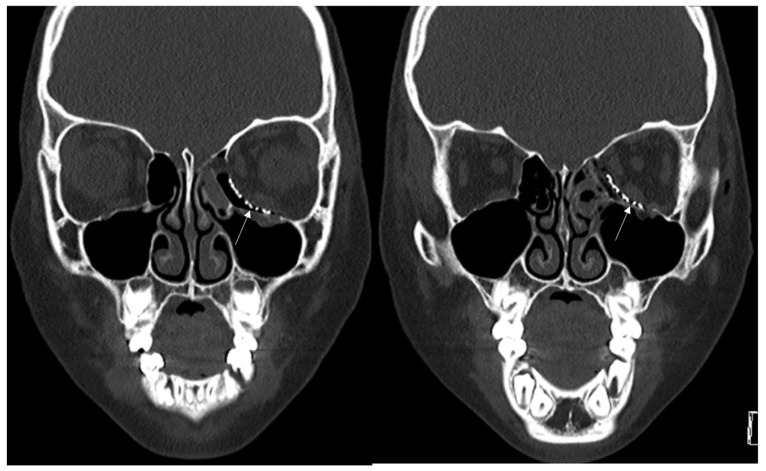
Case 1. Follow up facial CT finding (coronal view) of the same patient. The titanium-reinforced porous polyethylene (TR-PPE) plate placed on the inferomedial wall using a single screw at the inferior orbital rim. (White arrow).

An 8-year-old boy came to our emergency room with nausea, vomiting, bradycardia, and lethargy following a left eyeball trauma hit by a baseball. The patient complained of severe pain and discomfort with eyeball movement (Figure 1). We surgically approached the inferomedial orbital wall fracture on the same day of the trauma. After the subciliary incision, the fracture was exposed. The titanium-reinforced porous polyethylene (TR-PPE) plate was placed on the fracture site using a single screw at the inferior orbital rim (Figure 2). The oculocardiac reflex was entirely gone immediately after surgery. The patient was discharged on postoperative day 2 with significantly improved pain and discomfort.

### 3.2. Case 2

**Figure 3:**
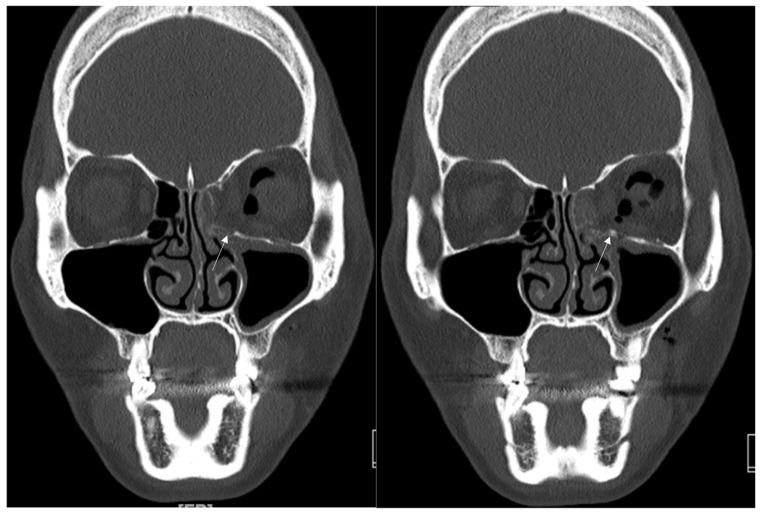
Case 2. Preoperative facial CT scan (coronal view) of the 48-year-old female patient with left inferomedial orbital wall fracture. The titanium-reinforced porous polyethylene (TR-PPE) plate was placed on the inferomedial orbital wall using a single screw at the inferior orbital rim. (White arrow).

**Figure 4:**
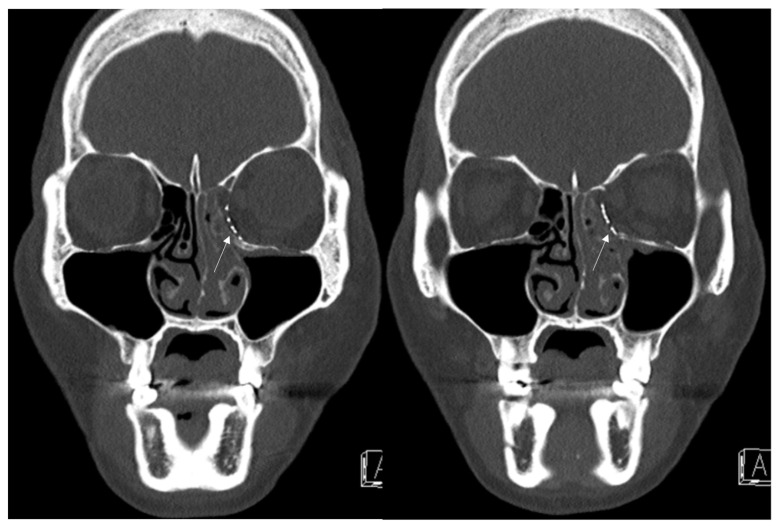
Case 2. Follow up facial CT finding (coronal view) of the same patient. The titanium-reinforced porous polyethylene (TR-PPE) plate was placed on the inferomedial orbital wall using a single screw at the inferior orbital rim. (White arrow).

A 48-year-old female patient came to our clinic with left eyeball swelling and ecchymosis caused by a car accident. The patient complained of pain and discomfort with eyeball movement (Figure 3). We surgically approached the medial orbital wall fracture seven days after the accident. After the subciliary incision, the fracture was exposed. The titanium-reinforced porous polyethylene (TR-PPE) plate was placed on the fracture site using a single screw at the inferior orbital rim (Figure 4). The patient was discharged on postoperative day 2 with significantly improved pain and discomfort.

### 3.3. Case 3

**Figure 5:**
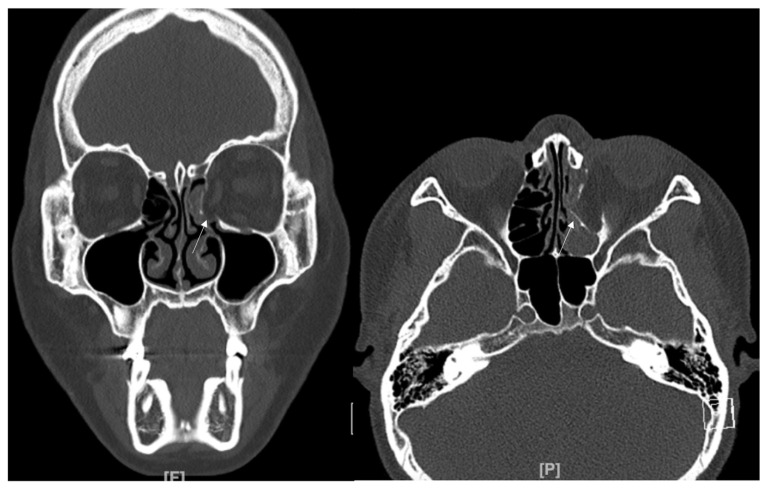
Case 3. Preoperative facial CT scan (coronal and axial view) of the 34-year-old female patient with left medial orbital wall fracture (white arrow). The fracture sites were designated with white arrows.

**Figure 6:**
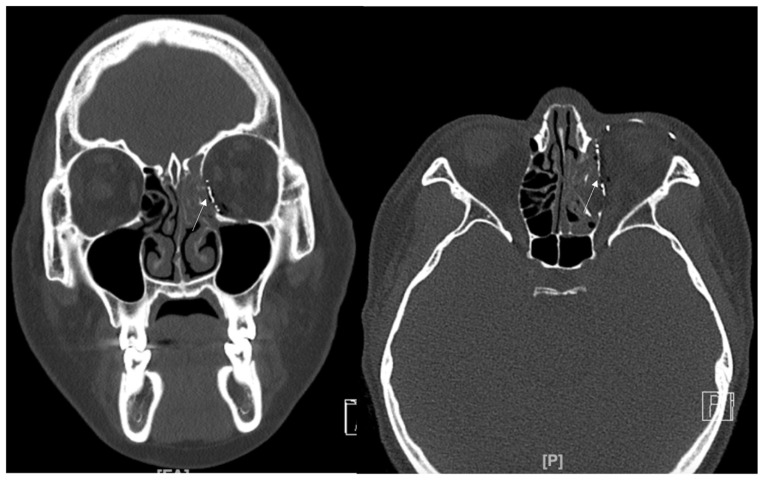
Case 3. Follow-up facial CT finding (coronal and axial view) of the same patient. The titanium-reinforced porous polyethylene (TR-PPE) plate was placed on the inferomedial orbital wall using a single screw at the inferior orbital rim. (White arrow).

A 34-year-old female patient came to our clinic with left eyeball swelling and ecchymosis caused by a direct trauma to the eyeball. The patient complained of pain and discomfort with eyeball movement (Figure 5). We surgically approached the medial orbital wall fracture two days after the accident. After the subciliary incision, the fracture was exposed. The titanium-reinforced porous polyethylene (TR-PPE) plate was placed on the fracture site using a single screw at the inferior orbital rim (Figure 6). The patient was discharged on postoperative day 2 with significantly improved pain and discomfort.

### 3.4. Case 4

**Figure 7:**
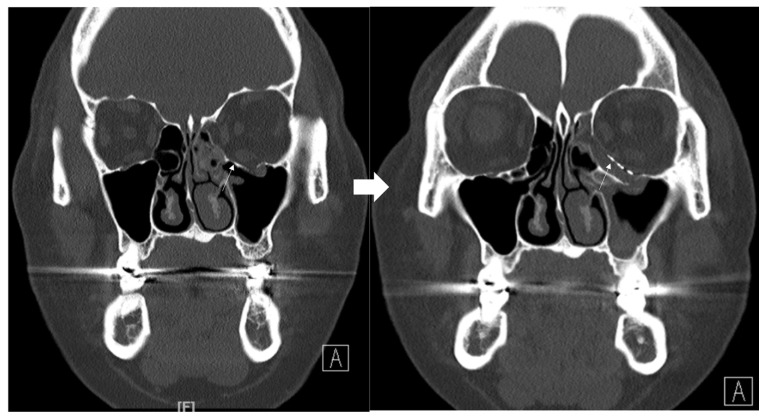
Case 4. Preoperative facial CT scan (coronal view) of the 51-year-old female patient with left inferomedial orbital wall fracture (white arrow) and follow-up facial CT finding (coronal and axial view) of the same patient.

A 42-year-old female patient came to our clinic with nausea and vomiting following a left eyeball trauma. The patient complained of pain and discomfort with eyeball movement. We surgically approached the inferomedial orbital wall fracture one day after the accident. After the subciliary incision, the fracture was exposed. The titanium-reinforced porous polyethylene (TR-PPE) plate and acellular dermal matrix beneath the plate were placed on the fracture site using a single screw at the inferior orbital rim (Figure 7). The oculocardiac reflex was entirely gone immediately after surgery. The patient was discharged on postoperative day 2 with significantly improved pain and discomfort.

## 4. Discussion

In this study, we share our successful treatment of medial orbital wall fractures or inferomedial wall fractures using a TR-PPE plate that is reinforced with titanium. This material is relatively stiff and holds its shape just as it was molded during surgery. Owing to these characteristics, we used titanium-reinforced porous polyethylene (TR-PPE) plates in extensive medial orbital walls or inferomedial wall fractures involving IOS requiring rigid fixation of the plate at the inferior orbital rim.

There are different materials available for reconstructing the orbital wall after a blowout fracture, but based on our experience, titanium-reinforced porous polyethylene (TR-PPE) plate has the strongest qualities [11,12,13,14]. If the inferomedial wall fracture is severe, additional materials may be used in combination, but the primary reconstruction should always be done with a rigid and fixable material.

The transcaruncular approach is now a popular incision method for treating pure medial wall orbital fractures; this is the same in our clinical practice. However, this incision is mostly utilized for pure medial wall fractures for us, particularly those affecting the middle to upper portion of the medial wall. If the medial wall fracture affects the lower part of it, including IOS, it is crucial to fix it securely, avoiding any possible implant migration or mispositioning after surgery. For extensive inferomedial wall fractures, several authors have utilized the extended transconjunctival approach and transcarucular incision to achieve a favorable outcome [7]. Others used a simultaneous standard transconjunctival inferior fornix incision and a standard precaruncular incision [15].

Based on our analysis, we experienced two cases of oculocardiac reflex in extensive inferomedial wall fracture involving IOS (Cases 1 and 4). Typical presenting signs and symptoms include bradycardia, alterations in blood pressure, faintness, and nausea when any of the peripheral branches or the central component of the trigeminal nerve is stimulated [16]. As noted in the previous literature, a series of symptoms, such as nausea, vomiting, and bradycardia, appear to predict a true incarceration of extraocular muscles in orbital wall fractures. However, true entrapment is not always needed to elicit the oculocardiac reflex [17]. This is because extensive orbital wall fractures may result in substantial tension on the inferior rectus muscle to induce the oculocardiac reflex [18]. Two cases with those symptoms preoperatively in our cohort were immediately resolved after the successful reduction.

**Figure 8:**
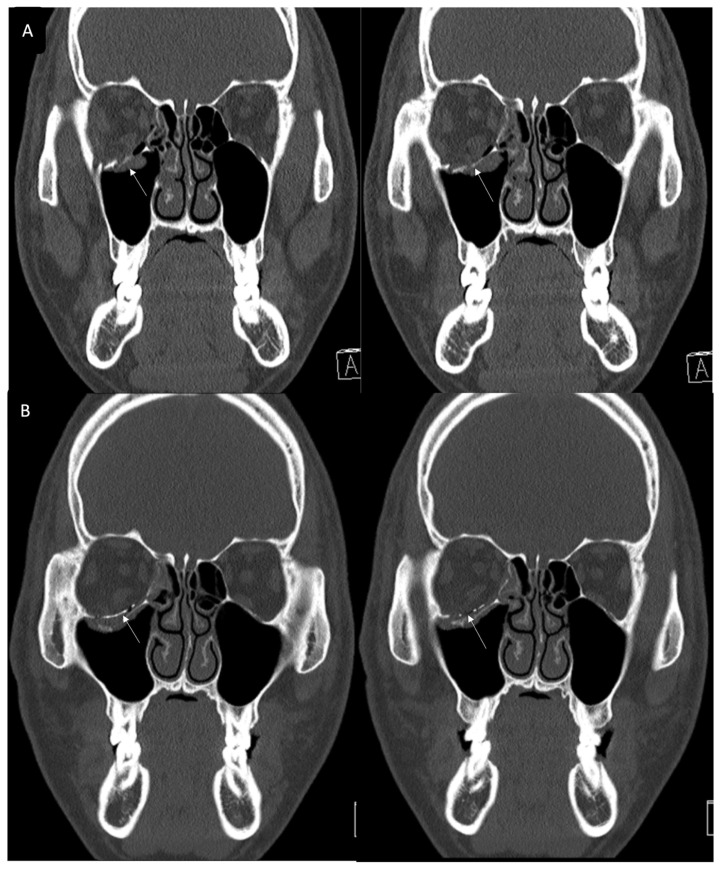
(**A**) A 29-year-old male patient came to our clinic with right eyeball swelling and ecchymosis caused by a direct trauma. The patient complained of pain and discomfort with eyeball movement. We surgically approached the inferomedial orbital wall fracture one day after the accident. After the subciliary incision, the fracture was exposed. (**B**) The titanium-reinforced porous polyethylene (TR-PPE) plate placed on the orbital floor using a single screw at the inferior orbital rim. The patient was discharged on postoperative day 2 with significantly improved pain and discomfort. The fracture sites were designated with white arrows. The titanium-reinforced porous polyethylene (TR-PPE) plate was placed on the inferomedial orbital wall using a single screw at the inferior orbital rim. (White arrow).

In this study, we excluded inferior wall fracture involving IOS. However, as shown in Figure 8, this inferior wall fracture with the involvement of IOS is also a good candidate for using titanium-reinforced porous polyethylene (TR-PPE) plates.

As suggested by Consorti et al. [19] very recently, the use of novel technology can greatly improve the accuracy of the reconstruction and achieve satisfactory clinical outcomes. They suggested the Computerized Operation Neuronavigated Surgery Orbital Recent Trauma (CONSORT) protocol, a workflow designed for the primary reconstruction of orbital fractures with customized mesh and intraoperative navigation.

## 5. Conclusions

Based on our retrospective analysis, it has been determined that among the 131 recorded cases of orbital fractures, 14 of them (or 10.7%) involved the inferomedial orbital strut. Navigation-guided reduction using titanium-reinforced porous polyethylene (TR-PPE) plates can lead to predictable, reliable, and excellent outcomes for treating orbital fractures involving the inferomedial orbital strut without complications.

## Figures and Tables

**Table 1:** Demographic and clinical characteristics of the patients (N = 131).

	Orbital Fractures (*n* = 131, 100%)	Orbital Fractures Involving IOS (*n* = 14, 10.7%)
Age (years)	37.76 ± 16.7	31.64 ± 14.6
Gender		
Male	92 (70.2%)	6 (42.9%)
Female	39 (29.8%)	8 (57.1%)
Affected side		
Left	65 (49.6%)	10 (71.4%)
Right	63 (48.1%)	16 (28.6%)
Bilateral	3 (2.3%)	0 (0%)

## Data Availability

The data presented in this study are available on request from the corresponding authors. Publicly data sharing is not applicable to this article due to privacy policy.

## Supplementary Materials

The following supporting information can be downloaded at: https://www.mdpi.com/article/10.3390/jcm12154968/s1, Figure S1: The navigation system used in a blowout fracture; Figure S2: The navigation system used in a blowout fracture; Figure S3: Intraoperative appearance immediately after reduction of left medial blowout fracture involving IOS. The black arrow designates inserted single titanium-reinforced porous polyethylene (TR-PPE) plate. The white arrow indicates an absorbable screw, the yellow arrow indicates intact inferior oblique muscle, which usually acts as a reference point, blue arrow indicates the navigation system.

## References

[1] Shim W.S., Jung H.J. (2019). Management of Orbital Blowout Fractures: ENT Surgeon’s Perspective. J. Rhinol..

[2] Jung E.H., Lee M.J., Cho B.-J. (2022). The Incidence and Risk Factors of Medial and Inferior Orbital Wall Fractures in Korea: A Nationwide Cohort Study. J. Clin. Med..

[3] Lim N.K., Kang D.H., Oh S.A., Gu J.H. (2015). Orbital Wall Restoring Surgery for Inferomedial Blowout Fracture. J. Craniofacial Surg..

[4] Park J., Jo S., Choi H.Y. (2022). Clinical Results According to Inferior Oblique Manipulation in Patients with Inferomedial Blowout Fracture Involving the Orbital Strut. Clin. Ophthalmol..

[5] Park T.H. (2023). Orbitalization of Ethmoidal Sinus With Stacked Cross-linked Acellular Dermal Matrix: A New Strategy to Reconstruct Medial Orbital Wall Fracture. J. Craniofac. Surg..

[6] Kim J.H., Lee I.G., Lee J.S., Oh D.Y., Jun Y.J., Rhie J.W., Shim J.H., Moon S.H. (2020). Restoration of the inferomedial orbital strut using a standardized three-dimensional printing implant. J. Anat..

[7] Jeong S.H., Moon K.C., Namgoong S., Dhong E.S., Han S.K. (2023). Anatomical Reconstruction of Extensive Inferomedial Blow-Out Fractures Involving the Inferomedial Orbital Strut Using a Single Fan-shaped Titanium-Reinforced Porous Polyethylene Plate. J. Craniofacial Surg..

[8] Blessing N.W., Rong A.J., Tse B.C., Erickson B.P., Lee B.W., Johnson T.E. (2021). Orbital Bony Reconstruction With Presized and Precontoured Porous Polyethylene-Titanium Implants. Ophthalmic Plast. Reconstr. Surg..

[9] Bourry M., Hardouin J.-B., Fauvel F., Corre P., Lebranchu P., Bertin H. (2021). Clinical evaluation of the efficacy of materials used for primary reconstruction of orbital floor defects: Meta-analysis. Head Neck.

[10] Xia L., Gao C., Gong X., Zhang Y., He Y., An J. (2023). Comparison of Postoperative Enophthalmos Between Fresh and Delayed Unilateral Orbital Fractures After Orbital Reconstruction With Titanium Mesh Using Computer-Assisted Navigation. J. Craniofacial Surg..

[11] Dvoracek L.A., Lee J.Y., Unadkat J.V., Lee Y.H., Thakrar D., Losee J.E., Goldstein J.A. (2021). Low-Cost, Three-Dimensionally-Printed, Anatomical Models for Optimization of Orbital Wall Reconstruction. Plast. Reconstr. Surg..

[12] Choi J.S., Oh S.Y., Shim H.S. (2019). Correction of post-traumatic enophthalmos with anatomical absorbable implant and iliac bone graft. Arch. Craniofacial Surg..

[13] Kim T.-H., Park I.-H., Hong S.-H., Eun S.-C. (2017). Sliced Costochondral Chip Grafts in Posttraumatic Enophthalmos Correction. J. Craniofacial Surg..

[14] Park T.H. (2023). Effectiveness of Cross-Linked Acellular Dermal Matrix to Correct Post-Traumatic Enophthalmos. J. Craniofacial Surg..

[15] Zhou G., Tu Y., Yu B., Wu W. (2021). Endoscopic repair of combined orbital floor and medial wall fractures involving the inferomedial strut. Eye.

[16] Brasileiro B.F., Sickels J.E.V., Cunningham L.L. (2020). Oculocardiac reflex in an adult with a trapdoor orbital floor fracture: Case report, literature review, and differential diagnosis. J. Korean Assoc. Oral Maxillofac. Surg..

[17] Toyohara Y., Mito N., Nakagawa S., Yoshimura K., Sunaga A. (2022). Asystole Due to Oculocardiac Reflex during Surgical Repair of an Orbital Blowout Fracture. Plast. Reconstr. Surg. Glob. Open.

[18] Al-Qattan M.M., Al-Qattan Y.M. (2021). “Trap Door” Orbital Floor Fractures in Adults: Are They Different from Pediatric Fractures?. Plast. Reconstr. Surg. Glob. Open.

[19] Consorti G., Betti E., Catarzi L. (2022). Customized and Navigated Primary Orbital Fracture Reconstruction: Computerized Operation Neuronavigated Surgery Orbital Recent Trauma (CONSORT) Protocol. J. Craniofacial Surg..

