# Supplementary material for: The Usefulness of the Navigation System to Reconstruct Orbital Wall Fractures Involving Inferomedial Orbital Strut
Source: J Clin Med. 2023 Jul 28;12(15):4968. doi: 10.3390/jcm12154968 (PMC10419887; doi:10.3390/jcm12154968)
Supplement: Supplementary file 1 [file jcm-12-04968-s001.zip › jcm-2469602-supplementary.pdf]

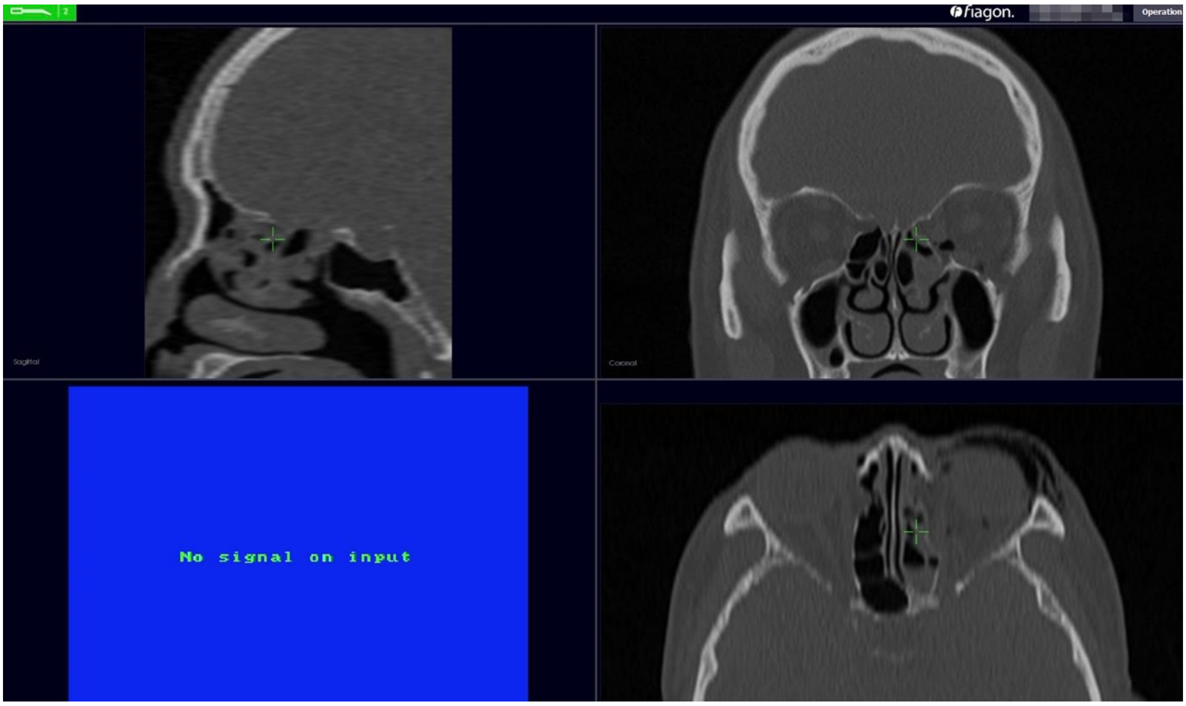

Figure S1. The navigation system used in a blowout fracture.

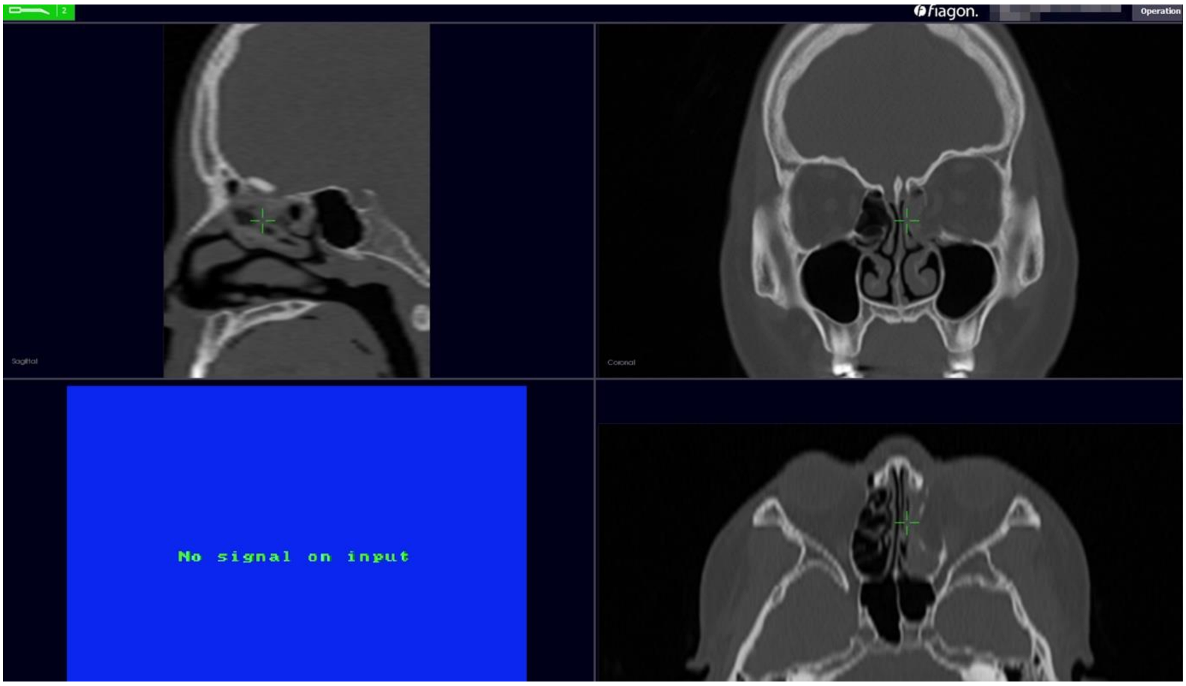

Figure S2. The navigation system used in a blowout fracture.

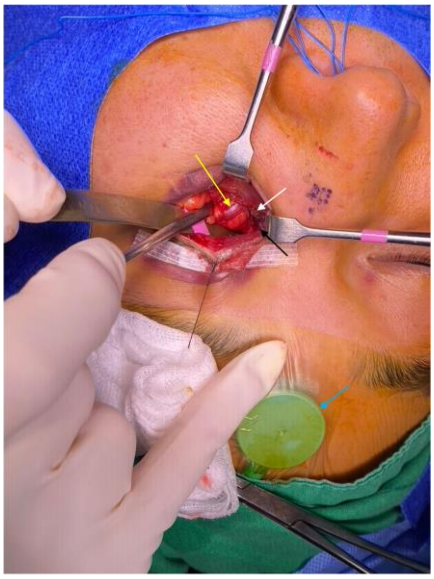

Figure S3. Intraoperative appearance immediately after reduction of left medial blowout fracture involving IOS. The black arrow designates inserted single titanium-reinforced porous polyethylene (TR-PPE) plate. The white arrow indicates an absorbable screw, the yellow arrow indicates intact inferior oblique muscle, which usually acts as a reference point, blue arrow indicates the navigation system.
